# Slt2 kinase of the cell wall integrity pathway is required for Fks2-specific echinocandin resistance in *Candida glabrata*

**DOI:** 10.1128/spectrum.03874-25

**Published:** 2026-08-10

**Authors:** Zubayeda Uddin, Saira Tahsin, Klara Valickova, Tengfei Long, Laura Fruhauf De Macedo, Gabrielle Popencuk, Yanan Zhao, Kelley R. Healey

**Affiliations:** 1 Department of Biology, William Paterson University 15665, Wayne, New Jersey, USA; 2 School of Pharmacy and Pharmaceutical Sciences, University at Buffalo 12292 , Buffalo, New York, USA; Seton Hall University, South Orange, New Jersey, USA

**Keywords:** *Candida glabrata*, echinocandin resistance, triazole resistance, cell wall integrity pathway, Slt2, *FKS1*, *FKS2*

## Abstract

*Candida glabrata* is an opportunistic yeast that causes invasive infections in immunocompromised individuals. Echinocandin antifungals are first-line agents in the treatment of invasive candidiasis and act by inhibiting fungal cell wall synthesis by targeting β-1,3-glucan synthase. Echinocandin resistance is primarily associated with mutations in *FKS1* or *FKS2*, which encode for the catalytic subunit (Fks1 or Fks2) of the targeted glucan synthase. The cell wall integrity (CWI) pathway, including Slt2 kinase, is known to mediate tolerance to antifungal drugs, but its role in resistance is not completely defined. Here, we observed that disruption of *SLT2* abrogated echinocandin resistance due to *FKS2*, but not *FKS1,* mutation in *C. glabrata*. As expected, reintroduction of plasmid-borne *SLT2* restored elevated MICs of *fks2* mutants. RT-qPCR revealed that loss of *SLT2* led to decreased *FKS2* expression levels in wild-type and echinocandin-resistant strains following echinocandin exposure. In conclusion, the CWI pathway and Slt2 comprise a primary control mechanism for *FKS2*-mediated echinocandin resistance in *C. glabrata*.

**IMPORTANCE:**

The higher rates of acquired antifungal resistance exhibited by *Candida glabrata* merit further understanding of resistance mechanisms including fungal regulation of drug target genes and enzymes. Here, we focused on a fungal tolerance pathway, the cell wall integrity pathway, and its requirement in *C. glabrata* echinocandin antifungal resistance. Interestingly, we found that targeting the pathway through disruption of a key protein, Slt2, reversed echinocandin resistance within one subset of resistant strains (*fks2* mutants) but not another (*fks1* mutants). Gene expression studies demonstrated the requirement of *SLT2* for full *FKS2* gene expression following echinocandin treatment. Overall, we discovered that Slt2 is specifically required for Fks2-mediated echinocandin resistance through partial regulation of *FKS2* expression. This study provides further insight into the regulation of drug target genes in *C. glabrata* and provides a possible therapeutic target for echinocandin-resistant infections caused by mutation of *FKS2*.

## OBSERVATION

*Candida glabrata* (also known as *Nakaseomyces glabratus*) has emerged as a significant concern due to its increasing global prevalence and high mortality rates, especially among patients with underlying conditions (e.g., 38%–45% in cancer patients [1]). Additionally, *C. glabrata* exhibits some of the highest rates of antifungal drug resistance (2–5) among all *Candida* species. Echinocandins and triazoles are the recommended first- and second-line antifungals, respectively, for invasive candidiasis and candidemia. The echinocandins (caspofungin, micafungin, anidulafungin, and rezafungin) target cell wall synthesis through inhibition of β-1,3-glucan synthase. In *C. glabrata*, the catalytic subunit of β-1,3-glucan synthase is encoded by two functionally redundant genes, *FKS1* and *FKS2*, and endpoint clinical resistance is primarily associated with specific mutations within either gene (6). Although acquired echinocandin resistance remains low (<1%) among other *Candida* species, 3% to 12% of *C. glabrata* strains now exhibit echinocandin resistance (2, 5). Triazoles (e.g., fluconazole, voriconazole, and itraconazole) target lanosterol 14α-demethylase, a key enzyme in the synthesis of ergosterol. In *C. glabrata*, clinical triazole resistance is primarily linked to gain-of-function amino acid substitutions within the transcription factor Pdr1 (7, 8). This, in turn, leads to the overexpression of ATP-binding cassette (ABC) transporters (i.e., *CDR1*, *CDR2/PDH1*, SNQ2) that can act as drug efflux pumps (9). Alarmingly, between 8% and 13% of *C. glabrata* strains in North America are currently considered fluconazole-resistant (5, 10). Based on these elevated rates of acquired resistance in *C. glabrata*, there is an urgent need for the identification of additional antifungal targets and/or the development of approaches that can overcome resistance to existing antifungals.

Upon exposure to stress, such as an antifungal drug, fungal cells activate various cellular mechanisms and pathways that can influence drug tolerance and resistance development (11). The cell wall integrity (CWI) pathway is a fungal tolerance pathway that is activated by various stressors, especially those that perturb the cell wall environment, and leads to the transcriptional activation of numerous cell wall related genes (12). This pathway is activated when plasma-membrane spanning sensors, including Slg1 (Wsc1 in *Saccharomyces cerevisiae*), cluster upon cell wall perturbation (13). This leads to the subsequent activation of various proteins including Protein Kinase C (Pkc1) and a MAP kinase (MAPK) cascade. The terminal MAPK, Slt2, activates specific transcription factors, including Swi4/6 and Rlm1, within the nucleus (14). While the activation of this pathway contributes to fungal tolerance of cell wall targeting compounds, including echinocandin antifungal drugs in *C. glabrata* (15–18), its significance in resistance has not been fully defined.

To provide further insight into the role of the CWI pathway on antifungal resistance in *C. glabrata*, we disrupted the *SLT2* gene with a *NAT1* marker in echinocandin-resistant *fks1* and *fks2* mutants and in triazole-resistant *pdr1* mutants (Table 1). The *fks1* (S629P), *fks2* (659delF and S663P), and *pdr1* (N768D and P927L) mutants were previously generated in strain ATCC 200989 (2001 *his*-/*trp*-/*ura*-) (19, 20), and strains of 2001 HTL (*his*-/*trp*-/*leu*-) and 2001 HTL ∆slt2::Ca*NAT1* were generously provided by K. Kuchler (21). We transformed each drug-resistant mutant strain with the ∆slt2::Ca*NAT1* cassette amplified from 2001 HTL ∆slt2. We also disrupted *SLT2* in the strain-matched (200989) wild-type background. Our gene disruption strategy is illustrated in Fig. S1, and related primers are listed in Table S1. Subsequent antifungal susceptibility testing was performed according to CLSI recommendations (22) and as previously described (19). All assays were repeated at least three times, and modal MICs were reported. With regard to triazole resistance, there was no effect on fluconazole or voriconazole susceptibilities following *SLT2* deletion in either *pdr1* mutant (Table 1). We obtained more interesting results when it came to echinocandin resistance. While there were no significant changes in micafungin or caspofungin MICs upon disruption of *SLT2* in an *fks1* mutant, we observed a complete reversal of resistance in two separate *fks2* mutants following *SLT2* deletion (Table 1). In fact, MICs decreased 32- to 128-fold in these *fks2* mutant strains.

**TABLE 1:** Disruption of *SLT2* reverses Fks2-mediated echinocandin resistance but not Fks1-mediated resistance nor triazole resistance

*C. glabrata* strain	24 h MIC (µg/mL)^*a*^	
	Micafungin	Caspofungin
2001 HTL wild type	0.03	0.03
2001 HTL Δslt2	0.016	0.016
200989 wild type	0.016	0.03
200989 Δslt2	0.008	0.016
200989 fks1-S629P	2	2
200989 fks1-S629P Δslt2	1	2
200989 fks2-S663P	1	2
200989 fks2-S663P Δslt2	0.016	0.03
200989 fks2-659delF	1	0.5
200989 fks2-659delF Δslt2	0.008	0.016

	Fluconazole	Voriconazole
2001 HTL wild type	16	2
2001 HTL Δslt2	16	2
200989 wild type	16	2
200989 Δslt2	16	1
200989 pdr1-N768D	128	16
200989 pdr1-N768D Δslt2	128	16
200989 pdr1-P927L	64	16
200989 pdr1-P927L Δslt2	64	16

^*a*^ Strains were tested in parallel a minimum of three times with modal MICs shown. Due to multple auxotrophies within the strains, assays were performed in YPD medium.

To confirm that the loss of *SLT2* and CWI pathway signaling caused the observed changes in echinocandin MICs, we complemented *SLT2*-disrupted strains with a plasmid-borne copy of the gene (p*SLT2*). A gap-repair approach was employed to clone *SLT2* onto plasmid pGRB2.0 (23) as illustrated in Fig. S2 (see Table S1 for primers). Strains were cultured and assayed in complete defined medium without uracil (SD-ura) (Sunrise Science Products, San Diego, CA, USA) to maintain selective pressure for plasmid retention and expression. We also expressed both empty vector and p*SLT2* in wild-type strains and each parental *fks2* mutant strain. As expected, the mere presence of either plasmid did not affect the elevated echinocandin MICs demonstrated by either resistant mutant (S663P or 659delF) (Table 2). In addition, each *fks2* mutant disrupted for *SLT2* exhibited the same reversal of resistance as described above despite the presence of empty vector. Importantly, we observed partial to full restoration of Fks2-mediated echinocandin resistance upon reintroduction of *SLT2* (4- to 16-fold increase in MIC) when compared to the same strain carrying empty vector (Table 2).

**TABLE 2:** Plasmid-borne expression of *SLT2* (p*SLT2*) induces complementary increases in echinocandin MICs in *fks2* mutant strains deleted for *SLT2*

*C. glabrata* strain^*a*^	24 h MIC (µg/mL)*^b^*	
	Micafungin	Caspofungin
200989 wild type + empty vector	0.12	0.06
200989 wild type + pSLT2	0.12	0.12
200989 Δslt2 + empty vector	0.06	0.06
200989 Δslt2 + pSLT2	0.12	0.12
200989 fks2-S663*P* + empty vector	2	>2
200989 fks2-S663*P* + pSLT2	2	>2
200989 fks2-S663P Δslt2 + empty vector	0.12	0.06
200989 fks2-S663P Δslt2 + pSLT2	1	1
200989 fks2-659delF + empty vector	1	2
200989 fks2-659delF + pSLT2	1	2
200989 fks2-659delF Δslt2 + empty vector	0.12	0.12
200989 fks2-659delF Δslt2 + pSLT2	0.5	0.5

^*a*^ pGRB2.0 was used to clone and ectopically express *SLT2 *(pSLT2).

^*b*^ Strains were tested in parallel a minimum of three times with modal MICs shown. Strains were maintained and assayed in complete defined medium without uracil (SD-ura).

**Fig 1:**
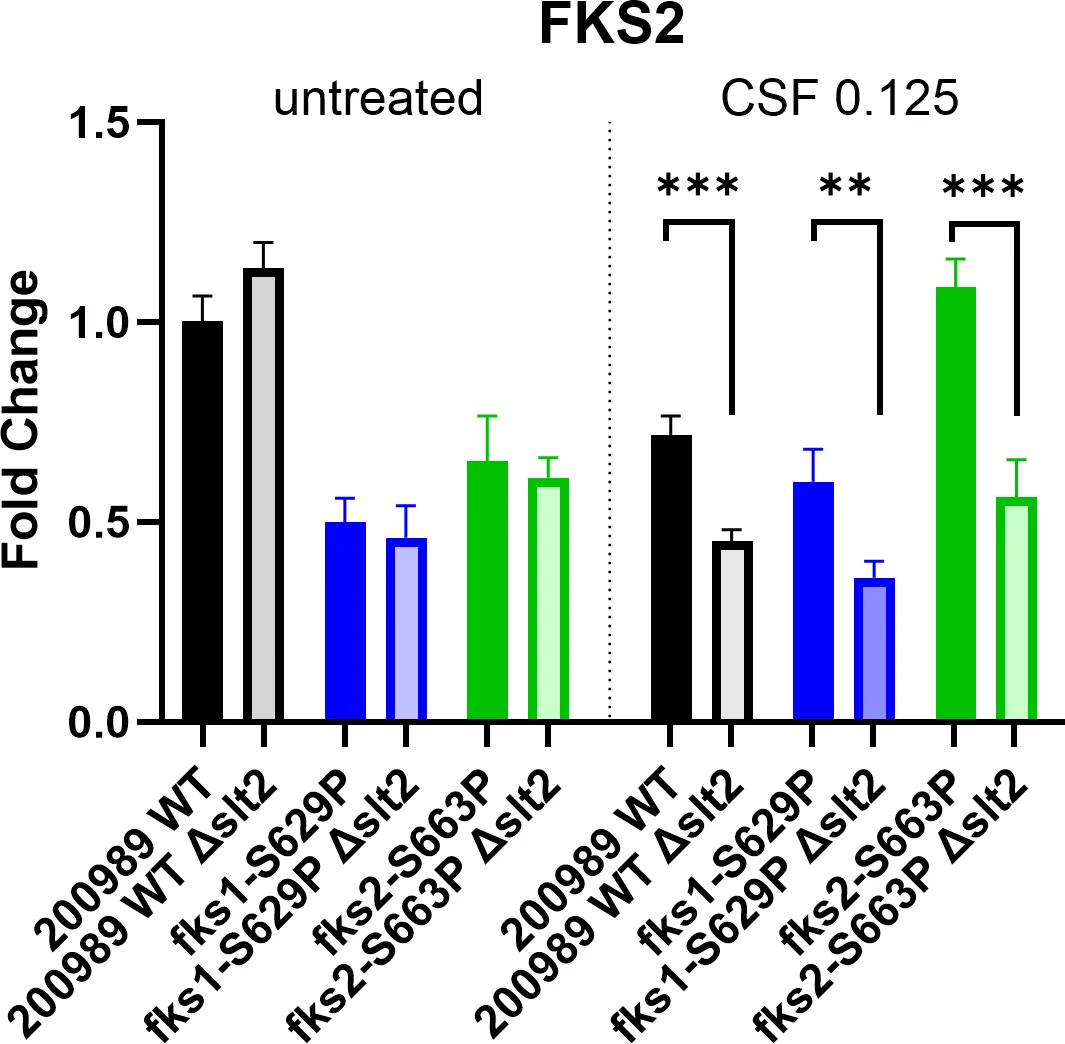
Strains deleted for *SLT2* exhibited consistent decreases in *FKS2* expression following echinocandin exposure. Fold change in expression levels of *FKS2* in untreated (left) or treated (right) cells were each compared to the untreated wild type (200989 WT) (means and standard deviations shown). Mid-log phase cultures of each strain were untreated or treated with 0.125 μg/mL of caspofungin [CSF] for 30 min, followed by RNA extraction and RT-qPCR. RNA was extracted from cells with MasterPure Yeast RNA purification kit (Biosearch Technologies) and the Power SYBR Green RNA-to-CT 1-Step Kit (ABI) was used for RT-qPCR. Reactions were run on an Applied Biosystems QuantStudio5 Real-time PCR System (Thermo Fisher Scientific). *PGK1* was used for normalization. Primers are listed in Table S1. At least two independent assays were performed for each strain, and each assay included three technical replicates. Statistical analysis was performed using paired Student’s *t*-test (two-tailed; **P < 0.01, ***P < 0.001).

We then set out to determine why deletion of *SLT2* resulted in a reversal of Fks2-mediated echinocandin resistance but not Fks1-mediated resistance. Research performed with *S. cerevisiae* has linked *FKS2* expression to CWI pathway signaling (13, 24). Here, we assessed *FKS1* and *FKS2* gene expression in wild-type and echinocandin-resistant strains with and without *SLT2*. In addition, we exposed cells to echinocandin drug to further activate the cell wall stress response and CWI signaling. Cultures grown to mid-log phase were untreated or treated with 0.125 μg/mL of caspofungin for 30 min as in reference 25, followed by RNA extraction and RT-qPCR. This concentration of drug represents 4× the MIC of wild-type strains. The exposure time and drug concentration were chosen to ensure the survival of enough *slt2Δ* cells for analysis and to specifically determine the influence of *SLT2* on *FKS* expression in response to echinocandin drug pressure. The relative quantification in gene expression was determined by the 2^−ΔΔCt^ method as previously described (26) using the house-keeping gene *PGK1* for normalization. Primers are listed in Table S1. Following echinocandin exposure, we observed significant decreases (40%–55%) in *FKS2* expression in all *slt2Δ* strains compared to their *SLT2*-containing parent strains (Fig. 1). In contrast, *SLT2* deletion had variable effects on *FKS1* expression (Fig. S3). The wild-type and *fks2* mutant strains deleted for *SLT2* exhibited modest increases (1.2- to 1.4-fold) in *FKS1* expression under echinocandin pressure (Fig. S3), likely reflecting compensatory upregulation in response to reduced *FKS2* expression. However, the remaining mutant exhibited decreased *FKS1* expression, which may indicate an indirect association between Slt2 and *FKS1*, although this requires further investigation. Of note, previous studies (25, 27, 28) have reported increases in *FKS2* expression upon echinocandin exposure. We observed moderate increases (1.2- to 1.7-fold) in *FKS2* expression in the parental *fks1* and *fks2* mutants, but not in the wild-type strain, following caspofungin exposure (Fig. 1). This variation is likely due to the shorter exposure time and/or lower drug concentration used here compared to other studies. Nevertheless, these results suggest that Slt2 is specifically required for full *FKS2* expression in *C. glabrata* following echinocandin exposure.

*Candida* fungi contain multiple *FKS* paralogs (*FKS1*, *FKS2*, *FKS3*). In most species, the *FKS1* gene is essential and only *FKS1* mutations are associated with echinocandin resistance (6). A unique characteristic of *C. glabrata* (and a shared characteristic with *S. cerevisiae*) is the ability to express *FKS1* or *FKS2* as the catalytic subunit of β-1,3-glucan synthase and, therefore, exhibit echinocandin resistance from mutation of either gene (6). While *FKS1* and *FKS2* are described as functionally redundant in *C. glabrata*, it has become clear that their regulation is not. In fact, studies in *C. glabrata* have pointed to their differential regulation under certain stressful conditions, such as changes in pH (29, 30). With respect to antifungal susceptibility and resistance, we previously reported that the RNA-binding protein, Ssd1, was required for full *FKS1* gene and protein expression and Fks1-mediated, but not Fks2-mediated, echinocandin resistance (19). In addition, Vu and colleagues found that the transcription factor Upc2A regulates *FKS1*, but not *FKS2*, expression (31). A recent study also highlighted the role of the cell cycle and growth conditions as key factors in the regulation of *FKS1* and *FKS2* gene transcription (32).

The expression of the *FKS2* gene in *C. glabrata* and *S. cerevisiae* is also uniquely dependent upon calcineurin signaling (25, 33), and genetic or chemical targeting of calcineurin reverses Fks2-specific echinocandin resistance in *C. glabrata* (25, 34). One study reported increased expression of both *SLT2* and *FKS2* in *C. glabrata* cells following micafungin exposure (35). This increase in *FKS2* was partially, but not fully, impaired by the loss of calcineurin. Recent evidence suggests calcineurin regulates *FKS2* in a post-transcriptional manner in *C. glabrata* (32). Overall, it appears that both the calcineurin and CWI pathways have control over *FKS2* expression and/or *FKS2* transcript stability. Relatedly, studies have highlighted the interplay and cross-talk between the calcineurin and CWI pathways within other fungi (24, 36, 37), partly due to the dependence of calcineurin signaling on the expression of genes associated with cell wall integrity (38). These studies, along with our current one, reinforce the idea that *FKS2* has an increased role in stressful conditions, and its increased expression is dependent upon these tolerance pathways. Of note, Schwarzmüller and colleagues (21) reported that *C. glabrata slt2Δ* exhibited increased susceptibility to caspofungin but not to other cell wall perturbing agents, including Congo red and Calcofluor white. Additionally, they reported no or minor changes in triazole susceptibilities. In addition, we found that the deletion of *SLT2* within triazole-resistant strains of *C. glabrata* did not significantly alter fluconazole or voriconazole MICs (Table 1). Altogether, these data may indicate an echinocandin-specific stress response that controls *FKS2* expression via Slt2; however, the precise mechanistic relationship between the CWI pathway and *FKS* gene expression in *C. glabrata*, and possible connections to calcineurin signaling, requires further investigation.

Finally, the significance of Slt2 in echinocandin tolerance exhibited by *C. glabrata* has been investigated *in vivo* (18). This study found that *C. glabrata* yeast cells without *SLT2* were cleared more effectively from the mouse gastrointestinal tract following echinocandin treatment and led to a reduction in the emergence of resistant strains. Interestingly, there was only one *fks2* mutant isolated from mice that were colonized with the *slt2* knockout strain. Surprisingly, this isolate exhibited susceptibility to echinocandins when assayed *in vitro*. Our current study explains this unexpected susceptibility and provides a possible target (CWI pathway/Slt2) to not only reduce resistance rates, but to reverse resistance in infections caused by mutation of the *FKS2* gene.

## Data Availability

The data used to support the findings of this study are presented in the paper and its supplemental material and are available from the corresponding author upon request.

## SUPPLEMENTAL MATERIAL

Supplemental table and figures spectrum.03874-25-s0001.docx Table S1; Fig. S1 to S3.

ASM does not own the copyrights to Supplemental Material that may be linked to, or accessed through, an article. The authors have granted ASM a non-exclusive, world-wide license to publish the Supplemental Material files. Please contact the corresponding author directly for reuse.
