# Supplementary material for: Slt2 kinase of the cell wall integrity pathway is required for Fks2-specific echinocandin resistance in Candida glabrata
Source: Microbiol Spectr. 2026 Aug 10;14(9):e03874-25. doi: 10.1128/spectrum.03874-25 (PMC13532262; doi:10.1128/spectrum.03874-25)
Supplement: Supplementary file 1 [file spectrum.03874-25-s0001.docx]

**Supplementary table 1.** **Primers used in this study.**

**Primer name	Sequence (5’ to 3’)	Application**

CgSLT2u224F	GTTTCAGTGATTGCTAAGTGGG	*SLT2* gene deletion (∆slt2::Ca*NAT1*

“deletion cassette” amplification)

CgSLT2d205R	CCTCACTAAATGTTCAGGAAC	*SLT2* gene deletion (∆slt2::Ca*NAT1*

“deletion cassette” amplification)

CgSLT2u305F	GGACAATCACACTGATCACTG	Confirmation of *SLT2* deletion

(PCR and sequencing)

CaNAT1c164R	GGTGGATCAACTGGAACTTC	Confirmation of *SLT2* deletion

CgSLT2c380R /	TGCGCATCTGTTAGTGCTTGC	Confirmation of *SLT2* deletion;

SLT2-iR	Confirmation of pSLT2 presence

pGRB-SLT2F*	gtcgacggtatcgataagcttg	SLT2 gap-repair primer for cloning

atatcgaattcctgcagGTTTCA

GTGATTGCTAAGTGG

pGRB-SLT2R*	GTTCCTGAACATTTAGTGAGGGG	*SLT2* gap-repair primer for cloning

atccactagttctagagcggcc

gccaccgcggtggag

T7-F	gcgtaatacgactcactatagg	Confirmation of pSLT2 and

empty plasmid presence

T3-R	AAGCGCGCAATTAACCCTCAC	Confirmation of empty plasmid presence

FKS1-F	CCTCACTATGCTGAAAGAATT	qRT-PCR quantification

FKS1-R	GGTATGGATGATCAAGATCCT	qRT-PCR quantification

FKS2-F	ATATGTCTGGTTCGTCAACTCC	qRT-PCR quantification

FKS2-R	CTGATCCACAAAGTCCCGTT	qRT-PCR quantification

PGK1-F	CAAACGGTGAAAGAAACGAGAA	qRT-PCR quantification

PGK1-R	CTTTCTTGAACGACTGTGTCGG	qRT-PCR quantification

**____________________________________________________________________________**

*underlined nucleotides include primer overhang regions homologous to plasmid sequences

**Supplementary figure 1.** ***SLT2* disruption in echinocandin-resistant (*fks1* and *fks2*) and triazole-resistant (*pdr1*) mutants of *C. glabrata.*** **A)** PCR amplification and purification of the ∆slt2::*NAT1* “deletion cassette” from strain 2001 HTL ∆slt2, **B)** Transformation of *pdr1* (or *fks1* or *fks2*) mutant cells (red X = mutation), **C)** Colony PCR screen of transformants, **D)** Final PCR confirmation of disruption following genomic DNA isolation of transformants. Primer sequences are listed in **Table S1**.

**Supplementary figure 2.** **Strategy for cloning and expression of *SLT2* via gap-repair cloning.** *C. glabrata*-specific plasmid pGRB2.0 was linearized with *SmaI*. *SLT2* was PCR amplified from a wild-type strain with gap-repair primers that contained overhang regions homologous to each side of the *SmaI* cut site on the plasmid. Competent *C. glabrata* cells were co-transformed with linearized plasmid and purified PCR and selected on complete defined agar medium without uracil (SD-ura). The 200989 strains are auxotrophic for uracil production. p*SLT2*-transformed colonies were screened with primers that annealed to the plasmid backbone (T7-F) and the *SLT2* coding region (SLT2-iR). Colonies transformed with empty vector were screened with primers that each annealed to the plasmid backbone (T7-F and T3-R). All primers are listed in **Table S1**.

**Supplementary figure 3.** **Varying changes in *FKS1* expression levels in *slt2*-deficient strains following caspofungin (CSF) exposure.** Fold change in expression levels of *FKS1* in untreated (left) or treated (right) cells were each compared to the untreated wild type (200989 WT) (means and standard deviations shown). Experiment performed as described in the text and in the Figure 1 description. All primers are listed in **Table S1**. Statistical analysis was performed using paired Student’s t-test (two-tailed; ** p < 0.05, ** p < 0.01*).
